# Bisphosphonate related osteonecrosis of the jaws (BRONJ) in osteoporotic males

**DOI:** 10.1186/s40064-016-3138-9

**Published:** 2016-09-01

**Authors:** Yong-Dae Kwon, Chae-Yoon Lee, Sung ok Hong, Yeon-Ah Lee, Joo-Young Ohe, Deog-Yoon Kim

**Affiliations:** 1 Department of Oral and Maxillofacial Surgery, Graduate School, Kyung Hee University, Seoul, Republic of Korea; 2 Division of Rheumatology, Department of Internal Medicine, Kyung Hee University School of Medicine, Seoul, Republic of Korea; 3 Department of Nuclear Medicine, Kyung Hee University School of Medicine, Seoul, 02447 Republic of Korea

**Keywords:** Bisphosphonate related osteonecrosis, ONJ, BRONJ

## Abstract

**Background:**

The purpose of this study was to describe the clinical characteristics of bisphosphonate related osteonecrosis of the jaws (BRONJ) in osteoporotic males.

**Methods:**

The medical records of BRONJ patients from 2007 to 2014 were reviewed. The data from only the male patients was extracted, and demographic data was collected and biochemical markers were measured.

**Results:**

11 Patients out of 210 (5 %) being males. Among the 11 patients, the indication of bisphosphonate (BP) was osteoporosis in 9 patients, and cancer in two. In one of the osteoporosis patients, osteoporosis was thought to be secondary to hypogonadism after testicular tumor resection. Serum c-terminal telopetide crosslink of type I collagen (s-CTX) values ranged from 60 to 165 pg/mL (mean: 84.6 ± 36.8, median: 70). Serum osteocalcin (s-OC) ranged from 0 to 5.06 ng/mL (mean: 1.83 ± 1.66, median: 1.5) and vitamin D ranged from 0 to 11.9 (mean: 5.02 ± 4.92, median: 3.5).

**Conclusion:**

BRONJ can be overlooked in male patients with osteoporosis. Although the incidence of BRONJ in males may be low, dentists should also check if their male patients are on osteoporosis treatment since osteoporosis in males can be manifested as a secondary disease to hypogonadism.

## Background

Osteoporosis is a substantially under-recognized disease in men and often remains untreated in many cases. Osteoporosis is considered to be found mostly in women because postmenopausal osteoporosis is the prevailing type of the disease. However, about 39 % of new osteoporotic fractures, which are the most frustrating complications of osteoporosis, were reported to have occurred in men by 2000 (Kaufman et al. 2013). The etiology of osteoporosis in males can be different from their female counterpart and the etiology is often secondary to some conditions.

Considering men have more morbidity and mortality after hip bone fractures than women do (Seeman 2002; Lambert et al. 2011), the adequate treatment of osteoporosis in men is also crucially important.

Since BRONJ has been well known to have a sexual predilection, clinicians and many general dentists usually ask only female patients whether they are on BP therapy. Therefore, history taking of BP therapy in male patients is rarely investigated. Like the current level of awareness of osteoporosis in males, BRONJ in males may be underestimated. Although the studies on BRONJ are rapidly increasing, little is known about BRONJ in males.

## Methods

From 2007 to 2014, all male patients that were diagnosed with BRONJ were enrolled in this study. BRONJ was diagnosed according to the criteria proposed by the American Association of Oral and Maxillofacial Surgeons (AAOMS) through radiographic and clinical examinations (Ruggiero et al. 2014). At risk category and stage 0 were not considered in our study. Only Stage 1, 2, 3 patients were inspected and grouped. Initial biochemical data were also included. The following clinical data were collected: age at diagnosis of BRONJ, gender, duration of BP use before diagnosis, underlying medical conditions contributing to osteoporosis, indications of BP, location and stage of BRONJ lesion, serum c-terminal telopeptide cross-link of type I collagen (s-CTX), serum osteocalcin (s-OC).

The study protocol was approved by the institutional review board at Kyung Hee University Dental Hospital (KHD IRB 1411-2).

## Results

**Fig. 1:**
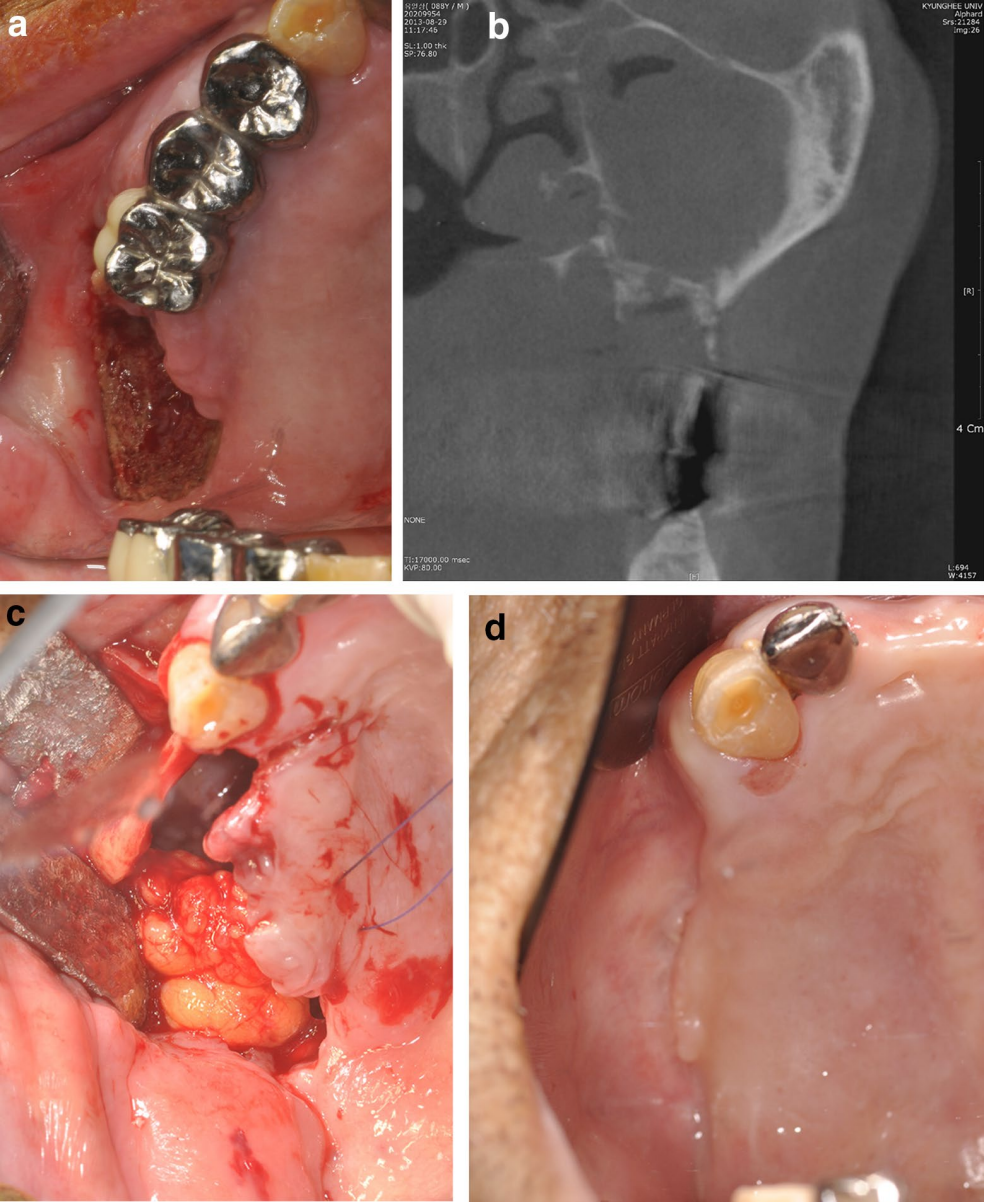
**a** Exposed necrotic bone surface on the right posterior maxillary region. (flipped from mirror image). **b** CBCT scan showed necrotic bone and heavy mucosal thickening obliterating the maxillary ostium. **c** Partial maxillectomy was carried out and buccal fat pad was mobilized to close the surgical wound. **d** Three months postoperatively, the wound was uneventfully healed. (flipped from mirror image)

The total number of the patients with BRONJ was 210 and the number of male patients was 11 (5 %). Among the patients with BRONJ, the indications of BP were osteoporosis in 9 patients, multiple myeloma in one and prostate cancer in one respectively. Ten patients presented one stage 2 lesion and one patient showed a stage 3 lesion (Fig. 1). One patient (No. 3) underwent total orchiectomy and chemotherapy because of testicular malignancy. Biochemical markers were checked (s-CTX, s-OC, Vitamin D) at baseline when BRONJ was diagnosed. s-CTX values ranged from 60 to 165 pg/mL (mean: 84.6 ± 36.8, median: 70). s-OC ranged from 0 to 5.06 ng/mL (mean: 1.83 ± 1.66, median: 1.5) and vitamin D ranged from 0 to 11.9 (mean: 5.02 ± 4.92, median: 3.5). The clinical data of the 9 BRONJ patients due to osteoporosis etiology are shown in Table 1.

**Table 1:** Demographic data of the male patients with BRONJ in this study

No.	Age	Stage/location	Contributing factor to osteoporosis	BP	Duration (years)	s-CTX^a^ (pg/mL)	S-OC^b^ (ng/mL)	Vit. D
1	77	2b/Mn	Primary	Risedronate	2	60	1.6	1.6
2	89	2/Mn	Primary	Alendronate	1	N/A	N/A	N/A
3	88	3/Mx	Secondary to hypogonadism^c^	Alendronate	10	70	0	0
4	66	2/Mn	Primary	Alendronate	1	80	0.97	11
5	68	2/Mn	Primary	Alendronate	1.5	60	1.6	N/A
6	67	2a/Mx	Primary	Alendronate	4	70	1.4	11.9
7	87	2b/Mn	Primary	Risedronate	3	60	3.5	3.5
8	77	2/Mn	Primary	Alendronate	3	165	5.06	6.7
9	71	2b/Mn	Primary	Alendronate	3	112	0.5	0.5

^a^Serum c-terminal telopeptide cross-link of type I collagen

^b^Serum osteocalcin

^c^Diagnosed with testicular malignancy and underwent orchiectomy and chemotherapy

*Mn* mandible, *Mx* maxilla, *BP* bisphosphonate

Information on s-CTX and osteocalcin testings in this study

CTX; ECLIA, Elecsys β-CrossLaps, Roche Diagnostics, Germany, Ref. range: < 704 pg/mL in males at 50–70 years old. < 854 pg/mL in males older than 70. <583 pg/mL in premenopausal women and < 1008 pg/mL in postmenopausal women

Osteocalcin; ELISA, DIASource Diagnostics, Belgium, Ref. range: 5–25 ng/mL

## Discussion

Osteoporosis in men has been a commonly overlooked etiology; therefore overlooking BRONJ in men, unless there are oncologic indications for BP medication, is even higher. A recent report from the National Nutritional Survey elucidated the level of recognition of osteoporosis in males to be 10.6 % and this figure was lower than that in females which was 24 % (Society 2014).

Also as many as one in four men over the age of 50 years will develop at least one osteoporotic fracture in their lifetime (Foundation). Osteoporosis in males can arise from geriatric reason and may be manifested as a secondary disease. Secondary causes for osteoporosis in men are as high as 65 %, while in women are only 20–40 % (Dy et al. 2011). Secondary osteoporosis can be a marker of systemic disease in the patient resulting from endocrine diseases such as diabetes mellitus, hyperparathyroidism, male hypogonadism; gastrointestinal disorders such as gastrectomy, celiac disease, inflammatory bowel disease; hematologic diseases such as myeloma, systemic mastocytosis; rheumatologic diseases such as rheumatoid arthritis, ankylosing spondylitis; and other reasons such as alcoholism, excessive smoking, and pulmonary emphysema (Ebeling 2008; Hofbauer et al. 2010). In our study, only 9 % of the patients (1 patient out of 11) had secondary osteoporosis. The other 8 patients had history of hypertension, brain aneurysm, tuberculosis, asthma, and rheumatic arthritis, but there was no evidence that these conditions were related to contributing into secondary osteoporosis. Untreated osteoporosis can trigger osteoporotic fractures leading to life-threatening conditions and the cumulative mortality rate can be higher in men than women (31 vs. 24.1 %) (Yoon et al. 2011). The treatment response to oral bisphosphonates in male osteoporosis is similar to that observed in postmenopausal osteoporosis, showing bisphosphonates as the choice of treatment for male osteoporosis (Kaufman et al. 2013). Because BP is one of the most common therapeutics for osteoporosis in males, such male patients may be at risk for BRONJ. BP therapy history taking is often neglected in a male patient set to have a surgical procedure involving alveolar bone. Even though there have been many papers on BRONJ since the first report was released, BRONJ in osteoporotic males has been rarely reported. Besides the long-term intake of BPs, concomitant risk factors can contribute to the development of BRONJ lesions. Those risk factors include corticosteroid therapy, diabetes, alcohol and smoking (Khosla et al. 2007). The existence of one or more BRONJ risk factors are expected to contribute to the early development of BRONJ but these results have yet to be statistically verified (Otto et al. 2011).

In our data, male BRONJ patients were 5 % of all BRONJ cases and only 4.3 % of all cases were BRONJ in osteoporotic males. Based on Otto et al. (2011), 36 % of their patients were males but this included BRONJ cases originating from oncologic use of BPs. Among BRONJ from oral BPs, 19 % were males (Otto et al. 2011). Because the indication of BP in BRONJ patients seems to be quite different across Asian countries (Urade et al. 2011; Lee et al. 2013) and western countries (Lazarovici et al. 2009; Otto et al. 2011; Walter et al. 2014), we found a clinical disparity between the 2 continents as well. BRONJ and atypical femur fracture (Shane et al. 2014) are the most frustrating complications of long-term BPs. According to a gender difference study of bisphosphonates-related complications, female gender has been thought to be a risk factor for atypical femur fractures but there was no significant gender difference for the development of BRONJ (Kharazmi et al. 2014).

Vitamin D and calcium supplements are recommended for the maintenance of bone mineral mass although conflicting data on the benefit still exist. One of the main secondary causes of osteoporosis in males is low calcium intake and vitamin D deficiency. In this study, the serum vitamin D level was quite lower than 30 ng/mL, which might cause impairment in bone remodeling, and failure to control osteoclastogenesis (Baldock et al. 2006). Therefore such conditions may precipitate osteoporosis, and vitamin D deficiency might be a risk factor for BRONJ as well.

Testosterone prevents bone loss and may increase bone mass in hypogonadism, although evidence is still lacking in long-term usage. In spite of the beneficial effect of testosterone on bone mass, ordinary osteoporosis therapeutics are usually selected (Boonen et al. 1997).

Several medications including BPs are believed to have a positive effect on BMD in the extreme event of hypogonadism due to chemical castration like one of the cases in the study (Smith 2007). It seems only reasonable to use BPs to prevent bone loss in men receiving androgen deprivation therapy. In prostate cancer patients, BP administration for these patients is quite well known even to general dentists, so specific history taking for BP intake is usually made. However, besides prostate cancer, other conditions inducing hypognoadism such as total orchiectomy and chemotherapy can also lead to osteoporosis and may increase the risk of BRONJ.

In very old age and hypogonadal conditions, BPs may be given to males in a long-term manner. Therefore, clinicians should ask patients if they have ever taken BP therapy regardless of their gender. Dental professions should be aware that male patients may be on BP therapy besides oncologic reasons therefore proper communication and referral should be established between the professions.
